# Something going on in Milan: a review of the 4th International PhD Student Cancer Conference

**DOI:** 10.3332/ecancer.2010.198

**Published:** 2010-11-10

**Authors:** C Segré

**Affiliations:** European School of Molecular Medicine, IFOM-IEO Campus, via Adamello 16, 20081, Milan, Italy

## Abstract

The *4th International PhD Student Cancer Conference* was held at the IFOM-IEO-Campus in Milan from 19–21 May 2010 http://www.semm.it/events_researchPast.php

The Conference covered many topics related to cancer, from basic biology to clinical aspects of the disease. All attendees presented their research, by either giving a talk or presenting a poster. This conference is an opportunity to introduce PhD students to top cancer research institutes across Europe.

The core participanting institutes included:

- European School of Molecular Medicine (SEMM)—IFOM-IEO Campus, Milan
- Beatson Institute for Cancer Research (BICR), Glasgow
- Cambridge Research Institute (CRI), Cambridge, UK
- MRC Gray Institute of Radiation Biology (GIROB), Oxford
- London Research Institute (LRI), London
- Paterson Institute for Cancer Research (PICR), Manchester
- The Netherlands Cancer Institute (NKI), Amsterdam

‘You organizers have crushed all my prejudices towards Italians. Congratulations, I enjoyed the conference immensely!’ Even if it might have sounded like rudeness for sure this was supposed to be a genuine compliment (at least, that’s how we took it), also considering that it was told by a guy who himself was the fusion of two usually antithetical concepts: fashion style and English nationality.

The year 2010 has marked an important event for Italian research in the international scientific panorama: the European School of Molecular Medicine (SEMM) had the honour to host the *4th International PhD Student Cancer Conference*, which was held from 19–21 May 2010 at the IFOM-IEO-Campus (http://www.semm.it/events_researchPast.php) in Milan.

The conference was attended by more than one hundred students, coming from a selection of cutting edge European institutes devoted to cancer research. The rationale behind it is the promotion of cooperation among young scientists across Europe to debate about science and to exchange ideas and experiences. But that is not all, it is also designed for PhD students to get in touch with other prestigious research centres and to create connections for future post docs or job experiences. And last but not least, it is a golden chance for penniless PhD students to spend a couple of extra days visiting a foreign country (this motivation will of course never be voiced to supervisors).

The network of participating institutes has a three-nation core, made up of the Netherlands Cancer Institute, the Italian European School of Molecular Medicine (SEMM) and five UK Cancer Research Institutes (The London Research Institute, The Cambridge Research Institute, The Beatson Institute for Cancer Research in Glasgow, The Patterson Institute for Cancer Research in Manchester and the MRC Gray Institute for Radiation Oncology and Biology in Oxford).

The conference is hosted and organised every year by one of the core institutes; the first was in Cambridge in 2007, Amsterdam in 2008 and London in 2009, this year was the turn of Milan.

In addition to the core institutes, PhD students from several other high-profile institutes are invited to attend the conference. This year participants applied from the Spanish National Cancer Centre (CNIO, Madrid), the German Cancer Research Centre (DKFZ, Heidelberg), the European Molecular Biology Labs (EMBL, Heidelberg) and the San Raffaele Institute (HSR, Milan). Moreover four ‘special guests’ from the National Centre for Biological Sciences of Bangalore (India) attended the conference in Milan. This represents a first step in widening the horizons beyond Europe into a global worldwide network of talented PhD students in life sciences.

The conference spread over two and a half days (Wednesday 19th to Friday 21st May) and touched on a broad spectrum of topics: from basic biology to development, from cancer therapies to modelling and top-down new generation global approaches. The final selection of presentations has been a tough task for us organisers (Chiara Segré, Federica Castellucci, Francesca Milanesi, Gianluca Varetti and Gian Maria Sarra Ferraris), due to the high scientific level of the abstracts submitted. In the end, 26 top students were chosen to give a 15-min oral presentation in one of eight sessions: Development & Differentiation, Cell Migration, Immunology & Cancer, Modelling & Large Scale approaches, Genome Instability, Signal Transduction, Cancer Genetics & Drug Resistance, Stem Cells in Biology and Cancer.

The scientific programme was further enriched by two scientific special sessions, held by Professor Pier Paolo di Fiore and Dr Giuseppe Testa, Principal Investigators at the IFOM-IEO-Campus and by a bioethical round table on human embryonic stem cell research moderated by Silvia Camporesi, a senior PhD student in the SEMM PhD Programme ‘Foundation of Life Science and their Bioethical Consequences’.

On top of everything, we had the pleasure of inviting, as keynote speakers, two leading European scientists in the fields of cancer invasion and biology of stem cells, respectively: Dr Peter Friedl from The Nijmegen Centre for Molecular Life (The Netherlands) and Professor Andreas Trumpp from The Heidelberg Institute for Stem Cell Technology and Experimental Medicine (Heidelberg).

All the student talks have distinguished themselves for the impressive quality of the science; an encouraging evidence of the high profile level of research carried out in Europe. It would be beyond the purposes of this report to summarise all 26 talks, which touched many different and specific topics. For further information, the Conference Abstract book with all the scientific content is available on the conference Web site (http://www.semm.it/events_researchPast.php). In what follows, the special sessions and the keynote lectures will be discussed in detail.

## 19 May 2010

### Special Scientific Session I: Giuseppe Testa, PhD (IEO, Milano)

#### Priming the epigenome in development and cancer

The conference had a flying start with a talk by Giuseppe Testa, Principal Investigator of the *Histone Methylation in Stem Cell Renewal and Lineage Commitment* Laboratory at the European Institute of Oncology, Milan.

It has been well established that the lineage commitment of a cell as well as the persistence of a specific differentiation stage is regulated by the modulation of its chromatin state through a code of epigenetic modifications of histone tails. Among the many modifications involved, methylation of histone H3 on lysine 4 (associated with active transcription) and on lysine 27 (associated with active transcription) reside at the centre of this network. Defined patterns of histone methylation are characteristic of different physiologic stages of differentiation, from pluripotency to terminal differentiation. Aberrant, stem cell like perturbations of the epigenetic patterns are common in cancer, where the cells reacquire a less differentiated, more proliferative phenotype. For example, repressive hypermethylation is found on the promoters of many tumour suppressor genes.

The work of Giuseppe’s laboratory is aimed at the decodification of the epigenetic code during both normal differentiation and oncogenesis. They have identified the existence of bivalent domains for both H3K4me3 and H3K27me3 on the promoter of different genes in undifferentiated, precursor cells. The resolution of the bivalent domain towards complete transcriptional activation (only H3K4me3) or transcriptional repression (only H3K27me3) leads cells to commitment and differentiation.

Giuseppe Testa’s laboratory is also at the forefront on another ‘hot field’ in modern biology; the elucidation of epigenetic changes driving the re-programming of somatic cells into induced pluripotent stem cells (IPs).

The ectopic expression of defined transcriptional factors was shown to induce the reacquisition of stem cell like properties [1], but the molecular details of this re-programming are largely unknown. A critical role is likely to be played by the histone methyl transferase Ezh2, a member of the polycomb group protein responsible for the trimethylation of lysine 27 on histone H3. This mechanistic relationship is being extensively investigated by Giuseppe Testa and his team.

Although the pattern of epigenetic markers and the chromatin state of IPs is more similar to that of an embryonic cell rather than a fully committed cell, IPs do not show the exact, equivalent pattern of epigenetic modifications as embryo-derived pluripotent cells. These striking observations not only complicate the picture of the mechanisms on the basis of cellular commitment and re-programming, but also raise relevant issues for therapeutic applications in medicine and in the public debate about this sensitive field of biological investigation.

One of the big promises and expectations of IPs is in fact the application in regenerative medicine and gene therapy. If it is possible to re-programme a somatic, committed cell to reacquire stem-like properties, it will be virtually possible to take adult cells from a patient, engineer them to treat the relevant pathology and re-programme them to be re-injected into the patient. This is perceived by public opinion as the magic wand that will solve once and for all the ethical dilemma of using human embryonic stem cells. But, in light of these new results, is this still so straightforward? Could IPs really substitute hES in every circumstance? It is not possible to have a clear answer yet, so further research in the IP and hES fields is required over the next decade. The ethical and social implications of IPs were also partially discussed during the Bioethics Round table on May 20th.

### Keynote Lecture I: Dr. Peter Friedl (The Nijmegen Centre for Molecular Life, The Netherlands)

#### Plasticity of cancer invasion and implications for therapy

It is not an easy job to keep the audience’s eyes fixed on the screen for more than one hour especially if you happen to be the last speaker of a densely scheduled day and much of your audience woke up at 4 pm (I hope you mean 4 am) to catch a flight, but Dr Peter Friedl succeeded in doing this.

He presented the results of his studies on cancer migration in living orthotopic fibrosarcoma xenograft mice, using the infrared-excited multiphoton microscopy (IR-MPM). With this avant-garde technology, it is possible to obtain three-dimensional images of tissues, resolving structures such as collagen fibres of the extracellular matrix, neural networks and blood vessels in living animals. It is really like opening a window on an organism and studying in real time the evolution of a biological phenomenon. Dr Friedl and his team injected tumour cells into recipient mice and applied IR-MPM to study how malignant cells start to migrate from the primary mass to metastatise.

The classical paradigm of metastatic onset and invasion is the so-called epithelial to mesenchymal transition, which defines the process through which cells acquire the ability to actively migrate through the extracellular environment. Cells can migrate in a dispersed, amoeboid way, or (as Friedl suggests) in a collective way. In his model cells at the front works as pacesetters in secreting collagenase, and a strand of many other cells follow as synchronously as a super-cell.

Interestingly, Dr Friedl clearly showed how in the living tissues tumour cells branched from the central mass and migrate as multicellular structures following pre-existing ‘highways’, such as tissue blood vessels (but, interestingly, not along neo-tumour-induced blood vessels). Cells can move with a speed of up to 200 μm per day and are also actively proliferating.

The IR-MPM was then used to study the response to radiation therapy. Strikingly, the perivascular, migrating cells were resistant to the treatment, while the vast majority of resting cells of the primary mass underwent apoptosis. Only the concomitant ablation of ß1 and ß3 integrins, which are very well characterised molecular actors in cellular migrations, was able to sensitise invasive cells to radiation, suggesting a central role of integrin-dependent signalling in response to radiotherapy.

A preliminary, combinatorial treatment with antibodies against integrin ß1 and subsequent irradiation was shown to be the only way to effectively eradicate all the migrating branches of the tumour mass *in vivo*.

Moreover, the notion that malignant cells prefer physiological microenvironments such as normal perivascular areas rather than new cancer-induced vessels is an essential concept for understanding the mechanisms of invasion and metastatisation and will be important for future cancer therapies.

## 20 May 2010

### Special Scientific Session II: Professor Pier Paolo Di Fiore (IFOM, Milano)

#### Endocytosis and re-cycling at the crossroads of signalling, attenuation, execution of polarized functions and cell fate determination

The *4th International PhD Student Cancer Conference* would have not been possible without the support of the European School of Molecular Medicine (SEMM), but the SEMM itself would not be a reality without the efforts of one of its principal co-founders, Professor Pier Paolo Di Fiore, whom we had the great pleasure to have as a special speaker. Pier Paolo Di Fiore is Principal Investigator in the *Endocytosis, Signalling and Cancer* Laboratory at IFOM, Milan.

Endocytosis of membrane receptor tyrosine kinases has traditionally been considered simply as a way for cells to attenuate long-term extracellular signalling. In recent years, many studies have shown that endocytosis is far more than that. Receptors are internalised into endosomes, which have been shown not to be simple ‘storage’ vesicles but complex structures where important decisions are taken. Receptors are degraded or recycled on the membrane according to the intensity of the extracellular stimuli; endosomes work as a platform for the integration of extracellular information into a complex network to execute important functions such as the establishment of cell polarity and cell motility. Among the many proteins and effectors involved, Numb is of particular interest since it is implied in the negative regulation of notch receptor signalling in actin and cytoskeletal dynamics.

Moreover, Numb is not only involved in endocytosis, but is also an important cell fate determinant in stem and progenitor cells.

Stem cells are undifferentiated cells able to give rise to a variety of differentiated cell types when induced by differentiation stimuli. They normally represent a minor part of cell populations in tissues and they are in a quiescent state respect to the more committed, proliferative cells.

Taking advantage from this concept, Prof. di Fiore together with Prof. Pier Giuseppe Pelicci have established an elegant experimental technique aimed at isolating and cultivating mammary stem cells (MSC).

Cells are stained with a red dye, PKH, which is retained in the cell membrane and is diluted in the daughter cells at every cell division. The more a cell is proliferating, the faster the dye will be diluted and the cells will loose the red colour. Cells that instead are quiescent, i.e. stem cells or precursors, will retain the dye and will appear red coloured, so that they and can be easily identified and purified.

Another key feature of stem cells is the ability to undergo not only symmetric division (which gives origin to two identical daughter cells more committed than the mother cell to linage determination), but also asymmetric division; one of the two cells retains the properties of the mother stem cells, thus maintaining the stem cell pool, while the other cell is committed to differentiation.

Using the PKH technique to enrich the stem cell content of cellular populations, Prof. di Fiore and his team discovered that Numb is differentially parted during asymmetric division in MSC: it is retained in the quiescent, stem cell while the other daughter cell acquires a differentiation fate and starts replicating symmetrically, loosing stem cell potential. Numb acts as a cell fate determinant because of its ability to silence the notch signalling pathway associated with differentiation and to positive regulate p53 in the MSC.

Fifty per cent of breast cancers show a decreased expression of numb, which is associated with a poor prognosis [2]. The findings that numb affects the regulation of the mammary stem cell compartment will likely have a great clinical relevance and will help to design more specific and effective pharmacological treatments.

### Special Session III-Bioethics round table: Worldwide human embryonic stem cells policies: Who decides and on what grounds? Moderated by Silvia Camporesi (PhD student, in the SEMM PhD programme in Foundation of Life Science and Their Bioethical Consequences – FOLSATEC)

The FOLSATEC is a unique, multidisciplinary PhD programme open to students with a scientific or philosophical background. It aims to create specialised scholars in the fields of ethical analysis and relationships between biomedicine and society. We could not miss the chance to include an interactive round table on bioethics in the conference programme.

Silvia Camporesi, a Senior FOLSATEC PhD student, gave a general but exhaustive summary of the current bioethical debate regarding research on human embryonic stem cells (hES cells). All the classical arguments against the use of hES cells were analysed, followed by counterarguments. The second part of the session was focused on an overview of the different hES cell policies in various countries.

The first argument used by opposers of hES cells is the so-called wisdom of repulsion enunciated by the American bioethicist Leon Kass, Chair of the US President’s Council on Bioethics from year 2002–2005, during the George W Bush administration.

According to him, the innate sense of repulsion towards something is the ‘expression of a deep wisdom, beyond reason’s power to fully articulate it’. Silvia replied with the words of one of the leading contemporary bioethicists, John Harris, Director of the Research Centre for the Institute for Science, Ethics and Innovation in Manchester (UK). It is not reasonable to use an ‘emotional, olfactory argument’ into a rigorous and logic debate. Moreover, it should be remembered that the ‘wisdom of repulsion’ has been used in the past to morally justify a presumed superiority of race and sex, which then science demonstrated to be totally groundless.

Another strong argument always proposed against the use of hES is the possibility to completely substitute them with IPs. But as Giuseppe Testa pointed out in his talk, IPs and ES cells are not identical. Moreover, the know-how that brought to the technology of IPs was mainly gained through research on embryonic stem cells. The most sensible approach will then be to pursue parallel research in both IPs and hES fields and exchange knowledge and technologies.

In the second part of the session, the current policies regarding hES of United States, United Kingdom, Germany and Italy were compared; the emerging picture was very heterogeneous and confusing, with peaks of depressing hypocrisy such as in the case of Italy, where the research is banned on hES created within the national boundaries but it can be done on hES cells lines imported from abroad.

The third part was dedicated to debate the illustrated topics with the audience; two main interesting considerations emerged. First, the different moral status societies give to embryonic stem cells on the basis of the ‘specie of origin’. There are in general no moral complaints about the use of animal embryonic stem cells, such as murine ones. This could be due, in part, to an innate ‘sense of empathy’ that we have towards something we perceive as being human, or having the potential to become human. In other words, our vision of the world is anthropo-centric and not mouse-centric; but is this argument strong enough from a logic and rational perspective to be used against human stem cell research? Is this argument strong enough to bypass all the possible good applications (such as regenerative medicine) that could arise from knowledge acquired in the hES cell research field?

Of course, we could not give a definitive answer; the debate is still open and it will likely be a ‘hot topic’ not only for the scientific and ethical communities but for democratic societies worldwide for many years to come.

The second issue of debate sprouted from the comparison of stem cell policies around the world. Everyone was convinced that the most urgent issue, beyond all the different approaches to the problem, is the realisation of a clear, unambiguous and globally uniform legislation in terms of use and derivation of human embryonic stem cells.

## 21 May 2010

### Keynote Lecture II: Professor Andreas Trumpp (DKFZ and Heidelberg Institute for Stem Cell Technology and Experimental Medicine, Heidelberg)

#### Dormancy in normal and malignant stem cells

Since the enunciation of the ‘golden rule’ of cancer onset and progression by Hanahan and Weinberg at the beginning of the XXI century [3], another revolutionary concept has changed the way scientists and physicians look (or should look) at cancer in the last years. To paraphrase a famous quotation: ‘*All the malignant cells are tumoural, but some are more tumoural than others’* [4]. That is, the tumour can be seen as an anarchist tissue inside the normal ones, with a hierarchy of heterogeneous cells; a population of differentiated, though malignant, cells and a few, pluripotent cells sustaining the growth and expansion of the whole mass.

Cancer stem cells have been now identified in many types of cancers; Professors Pier Paolo di Fiore and Pier Giuseppe Pelicci recently isolated breast cancer stem cells and demonstrated that they are not only responsible for the onset and growth of breast cancers, but also for the aggressiveness of the tumour. Cancer tissues with a higher number of malignant stem cells are more aggressive than cancers with a low number [5].

But the picture is even more complex, as Professor Trumpp masterly showed during his lecture. In fact, not all the stem cells are active; a sub-population can enter a resting state, defined as ‘dormancy’. These cells can create a suitable microenvironment, a niche, both in the tissue of the primary tumour or in a metastatic tissue. This ‘lethargy-like’ condition can be maintained by stem cells for a long time, even years, waiting to be ‘awaken’ by stress or injury stimuli such as interferon alpha or LPS [6]. It is not hard to imagine the impact that this new discovery will have on the way cancer is approached as a disease. New-generation drugs and therapies should be not only targeted to cancer stem cells more than the main tumour mass, but they should ideally be able to interfere with the dormant, not active stem cells directly on them or affecting somehow the establishment and/or maintenance of their proper niche. From a clinical point of view, this could lead to the eventuality to extend the temporal threshold of ‘remission’ far longer the canonical 5 years, with practical implications for example on cancer patient follow-up during time. But the science of stem cell biology is dawning now and many studies and discoveries will likely come in the next decades.

Besides all the excellent science, there has also been time for relaxing and social events. On the evening of the 19th we enjoyed pizza and the warm, jasmine-smelling air of mid-May at the IFOM-IEO-Campus bar.

The 360° view of the Milan by night was the frame of the Social Dinner at the Skyline Restaurant on the evening of the 20th May.

But since science and research never stop, it is already time to look forward: the 5th edition of the *International PhD Student Cancer Conference* will take place at The Beatson Institute for Cancer Research in Glasgow from 15–17 June 2011 (http://www.beatson.gla.ac.uk/Student-Conference-2011.html). Eminent scientists such as Gerard Evan and Nobel Prize winner Sir Tim Hunt will be giving lectures, and everyone is looking forward to attending what has clearly become an established and fruitful tradition in the panorama of European science.
